# A model of *k*-mer surprisal to quantify local sequence information content surrounding splice regions

**DOI:** 10.7717/peerj.10063

**Published:** 2020-11-04

**Authors:** Sam Humphrey, Alastair Kerr, Magnus Rattray, Caroline Dive, Crispin J. Miller

**Affiliations:** 1 CRUK Manchester Institute Cancer Biomarker Centre, The University of Manchester, Manchester, United Kingdom; 2 CRUK Manchester Institute, CRUK Lung Cancer Centre of Excellence, Manchester, United Kingdom; 3 Division of Informatics, Imaging and Data Sciences, University of Manchester, Manchester, United Kingdom; 4 Computational Biology Group, CRUK Beatson Institute, Glasgow, United Kingdom; 5 Institute of Cancer Sciences, University of Glasgow, Glasgow, United Kingdom

**Keywords:** Information theory, Surprisal, Splicing, Entropy

## Abstract

Molecular sequences carry information. Analysis of sequence conservation between homologous loci is a proven approach with which to explore the information content of molecular sequences. This is often done using multiple sequence alignments to support comparisons between homologous loci. These methods therefore rely on sufficient underlying sequence similarity with which to construct a representative alignment. Here we describe a method using a formal metric of information, surprisal, to analyse biological sub-sequences without alignment constraints. We applied our model to the genomes of five different species to reveal similar patterns across a panel of eukaryotes. As the surprisal of a sub-sequence is inversely proportional to its occurrence within the genome, the optimal size of the sub-sequences was selected for each species under consideration. With the model optimized, we found a strong correlation between surprisal and CG dinucleotide usage. The utility of our model was tested by examining the sequences of genes known to undergo splicing. We demonstrate that our model can identify biological features of interest such as known donor and acceptor sites. Analysis across all annotated coding exon junctions in *Homo sapiens* reveals the information content of coding exons to be greater than the surrounding intron regions, a consequence of increased suppression of the CG dinucleotide in intronic space. Sequences within coding regions proximal to exon junctions exhibited novel patterns within DNA and coding mRNA that are not a function of the encoded amino acid sequence. Our findings are consistent with the presence of secondary information encoding features such as DNA and RNA binding sites, multiplexed through the coding sequence and independent of the information required to define the corresponding amino-acid sequence. We conclude that surprisal provides a complementary methodology with which to locate regions of interest in the genome, particularly in situations that lack an appropriate multiple sequence alignment.

## Rationale

An accepted point mutation in a protein is a replacement of one amino acid by another, accepted by natural selection (Dayhoff, Schwartz & Orcutt, 1978; Dayhoff, Barker & Hunt, 1983). Genomic regions conserved between species therefore constitute islands of evolutionary stability within the more rapidly evolving nucleic acid sequence, and thus represent loci where important features are encoded. These observations make it possible to study evolutionary processes by generating multiple sequence alignments that seek to characterise the genetic changes that occur over evolutionary timescales. Methods such as the MEME and DREME suite of tools (Bailey & Elkan, 1995; Bailey, 2011), identify significant encodings using statistically enriched motifs in sets of functionally related molecular sequences. Here we have developed a complementary method to identify important sequence encodings within molecular sequences. Our approach measures the information provided by sub-sequences surrounding individual loci and we have shown this method can identify important genomic features. This method is alignment free, and hence can be applied broadly across all sequences, irrespective of overall sequence similarity, and independent of the functional relationships that might be used to group them. The approach can also evaluate different types of molecular sequences such as coding sequences and amino acids. Further applications of this approach therefore include the analysis of seemingly unconnected genomic loci such as those harbouring single nucleotide variants (SNVs) or somatic mutations.

## Introduction

### Information theory

In 1948, Shannon linked the information content of a sequence of symbols, first described by Hartley, and entropy, a quantity used in thermodynamics (Shannon, 1948; Hartley, 1928; Gibbs, 1902). Shannon’s discoveries initially focussed on transmission of messages over noisy channels. These later became fundamental principles in information storage (MacKay, 2003). Shannon Entropy is a measure of the complexity of an ensemble *X*, of symbols *x*, where each symbol occurs with a probability *p*(*x*). The self-information associated with each symbol is called *surprisal* (Tribus, 1961) and is defined by:

(1) \begin{eqnarray*}S(x)=-{\log \nolimits }_{2}(p(x))\end{eqnarray*}

where *S*(*x*) is measured in bits. For the full ensemble of *n* symbols, the total information of the ensemble is the sum over all surprisals $I(X)={\mathop{\sum }\nolimits }_{i=1}^{n}S({x}_{i})$. The Shannon entropy is defined as the average information per symbol or the expectation value of all surprisals:

(2) \begin{eqnarray*}H(X)=E(S(x))=-\sum _{i=1}^{n}p({x}_{i}){\log \nolimits }_{2}(p({x}_{i}))\end{eqnarray*}

where ${\mathop{\sum }\nolimits }_{i=1}^{n}p({x}_{i})=1$ and *H*(*X*) is also measured in bits. A few years after this formulation, the structure of DNA and the first protein sequences were discovered (Sanger, 1952; Watson & Crick, 1953). These discoveries and further advances in biology enabled the application of information theory to biological sequences. The storage of biological information within ensembles of molecular sequences led to several investigations of the quantification of biological information and its association with with biological functions (Gatlin, 1966). The Central Dogma of molecular biology itself was first framed in terms of the “transfer of sequential information” (Crick, 1970; Cobb, 2017), and information theory continues to underpin our understanding of how the genome encodes genetic information (Pritišanac et al., 2019).

A DNA sequence can be represented using an alphabet corresponding to the individual nucleotides 𝔸 = {*A*, *C*, *G*, *T*}. Since each amino acid is defined by a tri-nucleotide, or codon, it is useful to consider nucleotide sequences using an alphabet of 64 symbols, one for every possible tri-nucleotide. Of these, 61 codons encode amino acids, and the remaining three correspond to stop codons. Similarly, protein sequences can be defined by an alphabet of 20 symbols, one for each amino acid. This decline in the number of possible symbols between coding DNA, RNA, and protein leads to a decline in the maximum amount of information that can be encoded at each level. Information theory provides a theoretical framework within which to quantify these differences (Yockey, 1974; Nemzer, 2017).

The reason why almost all organisms translate only 20 amino acids despite the ability to encode 61 possible codons has not been fully determined (Koonin & Novozhilov, 2009). However, the redundancy in the genetic code allows for additional information to be captured within a coding region beyond that required to define the amino acid sequence itself (Yockey, 2000; Itzkovitz, Hodis & Segal, 2010). It has been suggested that codon degeneracy and the structure of the codon table support functions in addition to the encoding of amino acids (Dayhoff, Schwartz & Orcutt, 1978; Henikoff & Henikoff, 1992; Itzkovitz & Alon, 2007; Berleant et al., 2009; Maraia & Iben, 2014). These include the description of splicing regulatory motifs (Lim & Burge, 2001; Zhang & Chasin, 2004; Wang & Burge, 2008), DNA binding sites that co-exist within the coding sequence (Melnik & Usatenko, 2014; Vinga, 2014; Shreif, Striegel & Periwal, 2015), and RNA secondary structure (Itzkovitz, Hodis & Segal, 2010). This additional information can be viewed as a separate signal containing non-coding information multiplexed through the protein coding sequence.

While the genetic code naturally leads to a focus on triplet sequences, other representations are possible, and different length sequences reveal different aspects of the genome. For example, the information associated with individual nucleotides can be used to identify the presence of motifs within an ensemble of short molecular sequences (Schneider et al., 1986). It is important to consider that information content shown in motif figures is usually represented as 2 − *H*(*X*) as the aim is to identify consistent nucleotides within the motif rather than diversity (Schneider & Stephens, 1990). Dinucleotides can also be used to represent DNA sequences as they are important in the specification of epigenetic modifications (CpG islands), binding sites, splice donors and acceptors. Alphabets representing molecular sub-sequences of length *k* (*k*-mers) have also been widely used in motif discovery (Castle et al., 2008; Bailey, 2011). Information theoretic approaches have previously been used to investigate biological features such as coding and non-coding regions, nucleosome positioning and DNA binding sites with a variety of methods and representations to quantify information (Koslicki, 2011; Vinga, 2014; Wu, Zhang & Mu, 2014).

### Splicing

Splicing is an essential mechanism in human cells performed by the spliceosome, a large ribonucleoprotein complex comprised of five small ribonucleoproteins at its core plus many other protein cofactors (Matera & Wang, 2014). The process of splicing involves precise removal of intron sequences from the transcribed RNA sequence. Each gene can express multiple mRNAs with different patterns of intron removal, such that exons in one mRNA may be part of an intron for another. Approximately 95% of genes are spliced in humans through this exquisitely regulated process, which is responsible for much of the diversity in the proteome (Lee & Rio, 2015; Sahebi et al., 2016). For a mechanistic review see Shi (2017). Exon splicing is determined by the binding of three key molecules: U1, splicing factor 1 (SF1) and the U2 auxiliary factor (U2AF) to the 5′ splice site, the branch point and 3′ splice site within the intron, respectively. The decision to include an exon within a transcript is generally made at the time of binding of these three molecules and is mediated by the use of Splicing Regulatory Elements (SREs)  (Sickmier et al., 2006; Diederichs et al., 2016; Saha et al., 2020). Some of these SREs are embedded within exons and can be viewed as loci where additional information is multiplexed along with the information required for amino acid sequence determination. Analysis of recurrent *k*-mers near splice junctions have already been shown to predict novel splice sites and sequences involved in splicing  (Lim & Burge, 2001; Zhang & Chasin, 2004; Fairbrother et al., 2004; Schwartz, Hall & Ast, 2009; Raponi et al., 2011; Ke et al., 2011; Erkelenz et al., 2014).

Here we have created a method whereby different biological sequences can be interrogated without requiring a direct multiple sequence alignment, or the need for sufficient sequence conservation with which to build that alignment. Using splice sites as a set of known biological features, we show that our model can quantify the information content of sequences at these regions irrespective of species.

## Methods

### Annotation

Genomic annotation for 5 different species *Homo sapiens*, *Mus musculus*, *Drosophila melanogaster*, *Danio rerio*, *Schizosaccharomyces pombe* was downloaded from Ensembl v99 (ftp://ftp.ensembl.org/pub/) (Cunningham et al., 2019). Genomic DNA, coding mRNA, and peptide files, were mapped against genomic annotation provided by the gene transfer format (.gtf) file. Only protein coding transcripts with GENCODE basic annotation were included in this analysis, which is defined as the set of 5′ and 3′ complete transcripts. Exon-intron boundaries and exon-exon junctions were independently generated using the gene transfer format annotation and sequences were aligned at splice sites.

Only *k*-mers with nucleotide symbols consisting fully of 𝔸_*nt*_ = {*A*, *C*, *G*, *T*} or 𝔸_*aa*_ = {*A*, *C*, *D*, *E*, *F*, *G*, *H*, *I*, *K*, *L*, *M*, *N*, *P*, *Q*, *R*, *S*, *T*, *V*, *W*, *Y*} for amino acids were used in this analysis. Since the majority of eukaryote genes have multiple transcripts per gene, with many exons shared between them, when evaluating coding sequences and amino acids, the *k*-mers from those shared exons would be duplicated if all transcripts were considered individually. We therefore impose a multiple transcript correction such that the genomic locations; chromosome, strand, start and end positions, for all *k*-mers must be unique. The repeated *k*-mers used by different transcripts at the same genomic locus are discarded. This method is also sensitive to *k*-mers spanning exon-exon junctions, since it maps the positions to genomic locations. The coding mRNA distribution contains all *k*-mers from within coding sequences only; introns, UTRs, and non-coding transcripts were excluded. Amino acid *k*-mers were reduced in length by a factor of 3, ( ${k}_{aa}= \frac{{k}_{nt}}{3} $), and their value was allocated to all three nucleotides in their corresponding codon when comparing with DNA and coding mRNA sequences.

### Probability of *k*-mer occurrences

The *k*-mers starting at every position in the DNA (both strands), all coding mRNAs, and all amino acid sequences were extracted, counted and recorded in frequency tables. These frequency tables were then used to identify the probability of the *k*-mer, *x*, occurring in the overall sequence

(3) \begin{eqnarray*}p(x)= \frac{counts(x)}{\sum _{x}counts(x)} \end{eqnarray*}

where ∑_*x*_*counts*(*x*) is equivalent to the total number of *k*-mers in the sequence, which corresponds to the sequence length minus *k* and any discarded sequences. Using this measure of *k*-mer probability, the surprisal for each *k*-mer can be calculated by Eq. (1) while the Shannon entropy for the total sequence can be found using Eq. (2).

### Choice of *k*-mer length

This estimate of *k*-mer probabilities (Eq.(3)) works well for small *k*, however for larger *k* this entropy calculation is limited due to the finite sample size of biological sequences (Herzel, Ebeling & Schmitt, 1994; Herzel & Große, 1995). Since the total number of possible *k*-mers in nucleotide space is 4^*k*_*nt*_^ and is 20^*k*_*aa*_^ in amino acid space, the *H. sapiens* DNA sequence is too small to contain every possible *k*-mer for *k*_*nt*_ ≥ 17 in DNA sequences and *k*_*aa*_ ≥ 6 for amino-acids. The *k*-mer distributions associated with these spaces are also skewed, such that some *k*-mers do not occur even for much smaller *k*. Here we refer to *k*-mers with zero occurrences as ‘nullomers’ (Hampikian & Andersen, 2007). The presence of these nullomers at short *k*, combined with the skew in the distribution, lowers the observed entropy *H*^*obs*^ of the sequence. This information loss can be quantified in terms of the sequence redundancy:

(4) \begin{eqnarray*}R=1- \frac{{H}^{obs}}{{H}^{max}} \end{eqnarray*}

where the largest possible entropy of the system, ${H}_{nt}^{max}=-{\log }_{2}({4}^{-{k}_{nt}})=2{k}_{nt}$ bits for nucleotide sequences and ${H}_{aa}^{max}=-{\log }_{2}(2{0}^{-{k}_{aa}})$ bits for amino acid sequences. Since *H*^*max*^ is dependent only on *k* whilst *H*^*obs*^ is limited by the length of the sequence, *R* increases as the total number of possible *k*-mers becomes greater than sequence size. This effect is also dependent on the uniformity of the *k*-mer distribution since *H* is maximised when all *k*-mers are equally likely.

**Figure 1:**
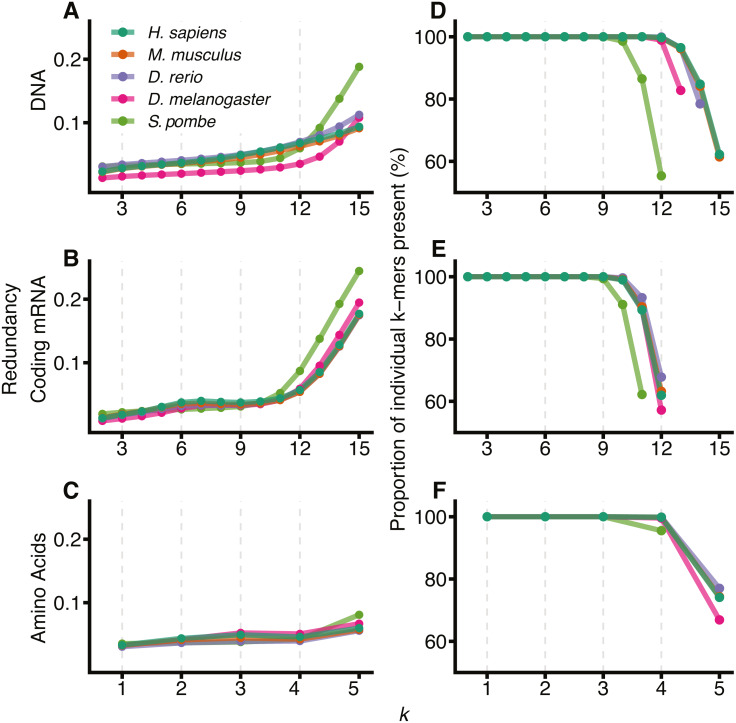
Visualising the sample size effect. The effect of increasing *k*-mer resolution for DNA (A, D), coding mRNA (B, E) and amino acid (C, F) sequences, where amino acids are considered at sizes $ \frac{k}{3} $. For each value of *k*, 2 ≤ *k* ≤ 15, the redundancy (Eq. (4)) of the region was calculated (A, B, C) along with the percentage of all possible *k*-mers observed at least once in the genome (D, E, F). D, E and F have been truncated at the point where the total possible number of *k*-mers, 4^*k*_*nt*_^ or 20^*k*_*aa*_^ exceeds the size of the region for that genome (∑_*x*_*counts*(*x*) for all *k*-mers, *x*).

Figure 1 shows the redundancy and the proportion of unique *k*-mers observed for 2 ≤ *k* ≤ 15 for the 5 different species: *H. sapiens*, *M. musculus*, *D. rerio*, *D. melanogaster* and *S. pombe*. As expected, all species show an increase in redundancy at larger *k* and a corresponding decrease in the number of *k*-mers represented at least once in the sequence. In DNA space (Figs. 1A and 1D), this effect becomes prominent at *k*_*nt*_ > 12. We therefore conclude that 12-mers are an appropriate size to model DNA sequences for all species except *S. pombe*, where they are too large relative to the size of the significantly smaller genome. We have therefore removed *S. pombe* from further analysis. Following similar reasoning, for comparisons between DNA, coding mRNA and amino acid sequences, we selected *k*_*nt*_ = 9 and *k*_*aa*_ = 3 to reflect the reduced amount of coding mRNA sequence relative to the DNA.

## Results

### Surprisal patterns across splice sites

**Figure 2:**
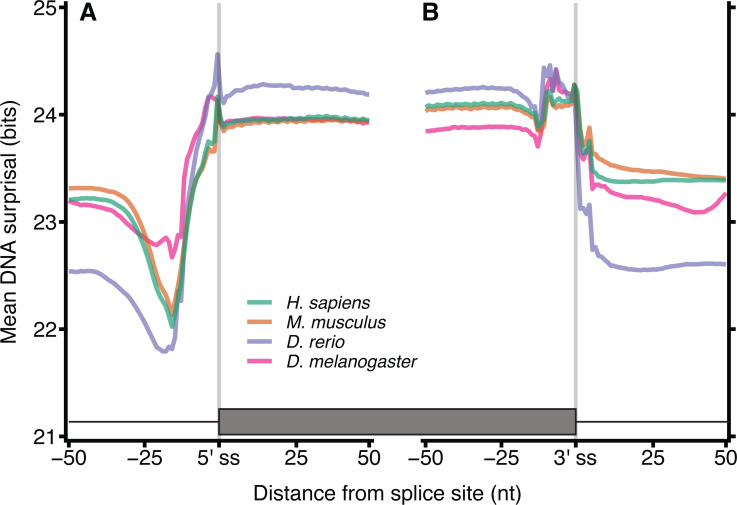
DNA Surprisal across exon-intron junctions identifies known features and reveals increased information within exons. For each of the 4 species, 12-mers were extracted at each position relative to the exon-intron junction for all protein coding exons. The mean surprisal of the 12-mers is plotted across the exon 5′ (A) and 3′ (B) splice site boundaries. Data were generated for all GENCODE basic, protein coding transcripts, using exon annotation downloaded from Ensembl (Cunningham et al., 2019). In both plots, the exon-intron junction occurs between positions −1 and 0, indicated by the grey line at position −0.5. Exon junctions falling within 100 nt of the transcript start or end site were removed.

Metazoan exon-intron junctions have known conserved features within introns. Downstream of the 3′ end of the vast majority of exons is the GT donor, and upstream of the 5′ end of the exon is a corresponding AG acceptor site. A pyrimidine rich region occurs approximately 4–20 nt upstream of the acceptor site and a conserved but location variable branch point occurs approximately 15–55 nt upstream of the acceptor site (Corvelo et al., 2010). These motifs make exon-intron junctions ideal candidates for testing our model. Figure 2 shows the mean of DNA 12-mer surprisal across all protein coding exon-intron junctions for each species. A consistent surprisal pattern is observed, with substantial changes in the mean surprisal at the position of the polypyrimidine tract and splice site motifs. This result is expected since the surprisal, and hence information, is inversely proportional to *k*-mer occurrence, and therefore surprisal decreases in the presence of common sequences motifs. Conversely, Fig. 2 also reveals that for all species, exons contain significantly greater information than introns near splice boundaries. Although apparently intuitive, it is surprising since exon sequences are constrained to contain only those sequence patterns capable of representing a functional protein, while intron sequences are under no such constraint. For example, *k*-mers featuring an in-frame stop codon are not permissible within a coding sequence. A naïve view of coding space, therefore, is one in which exon sequences are constructed from a subset of possible *k*-mers while intron sequences can be constructed from the entire repertoire of *k*-mers. This predicts that exons would be constructed from more common sequences, and that their surprisal would therefore be lower than that of the corresponding intronic space. By contrast, these data suggest a greater sequence constraint on intronic regions near splice junctions than similar proximal exon regions. This in turn implies that there are additional constraints for intronic sequences, such as those arising from the need to encode intronic SREs. It is tempting to speculate that these patterns result from a selection pressure that excludes certain valid coding sequences from intronic space. Importantly these patterns are robust against different values of *k*, demonstrating the generality of the model (Fig. S1). Here we show data for *k* = 12, since these have a lower coefficient of variation than those of smaller *k* (Fig. S2).

### Surprisal across exon junctions

**Figure 3:**
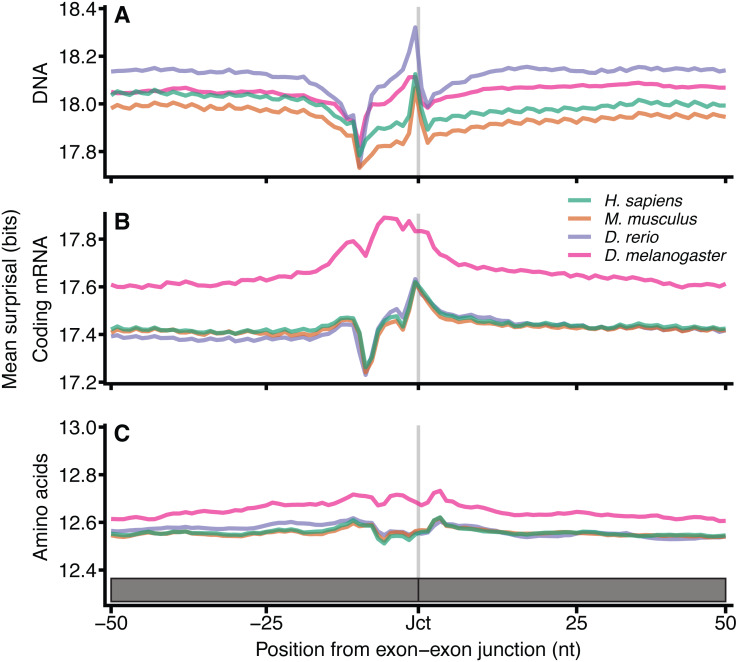
Surprisal across exon–exon junctions reveals non-coding information within coding sequences. For each of the 4 species, all 9-mers and amino acid 3-mers were extracted at each position relative to the exon-exon boundaries for all protein coding exons. The mean 9-mer DNA (A), 9-mer coding mRNA (B) and 3-mer amino acid (C) surprisal is plotted across the spliced exon boundaries centred on the exon junction. Data are plotted with the exon junction between positions −1 and 0 indicated by the grey line at position −0.5, and the *y* axes are a constant size, however the range is shifted dependent on the sequence type. In order to plot amino acid data on the same scale, the data for each exon junction were transformed into nucleotide space by repeating each amino surprisal value three times in succession. These data are in the positions by which they occur with respect to the exon junction in their respective phases. Source sequence data is as in Fig. 2, except that junctions within 100 nt of the translation start or end site were removed.

Since the distinct patterns in Fig. 2 arise from differences between coding and non-coding sequence spaces, we aimed to identify additional information encoded in the DNA and coding sequences beyond that which is required simply to define the encoded protein sequence. Protein coding exon-exon junctions were aligned at 9-mer resolution (amino acid 3-mers), with *k* = 9 chosen to account for the smaller sample size of coding sequences (Fig. 3). The mean surprisal for *H. sapiens* surrounding exon-exon junctions is 18.0 bits in DNA and 17.4 bits for coding mRNA, which as expected, is slightly lower due to the loss of in-frame stop codon sequences. Amino acid sequences show a significant reduction of approximately 1/3 information when compared to DNA sequences (12.6 bits). This is in keeping with previous work that considers the entropy of codons (Yockey, 1974). All other species considered here are consistent with *H. sapiens*, and show similar patterns in DNA surprisal across exon-exon junctions, with a constant difference dependent on the species. All species show a sharp decrease in surprisal at position −10 and sharp increase at position −1, 1nt upstream of the exon junction, consistent with Fig. 2. These 9-mers are created by the juxtaposition of the last nucleotide of the 5′ exon with the first 8 nucleotides of the 3′ exon. An interesting observation is that the most frequent dinucleotide across the junction for both exon-exon and exon-intron boundaries is GG, suggesting that the joining of the G in position −1 with the following 8-mer is driving this peak. For coding mRNA most species show a tight and consistent pattern similar to that of DNA surprisal, however *D. melanogaster* is a clear outlier, with a different pattern and a significantly higher information content. This is likely to be in part a consequence of the increased proportion of coding sequence in *D. melanogaster* (22.1% versus < 3% for the other species in Fig. 3).

The sharp decrease in surprisal is at position −9 for coding mRNA surprisal. When computed at other values of *k* (Fig. S3), these minima shift with *k*, indicating that the nucleotides driving this pattern are at the end of the *k*-mer, and suggesting that this pattern arises from the lesser conserved exonic 3 ′ AG terminal motif. There is little variation surrounding splice junctions for amino acid sequences which is expected since the information content encoded at these positions is expected to be a feature of splicing information, which is ahead of the translation process. This is consistent for other values of *k* (Fig. S3) and similarly to Fig. S2, the coefficient of variation is reduced for larger *k* (Fig. S4). Together these data strongly suggest that codon redundancy allows additional signals to exist within the DNA without significant impact on the amino acid sequence encoded through the same space.

### Effect of CG dinucleotides

**Figure 4:**
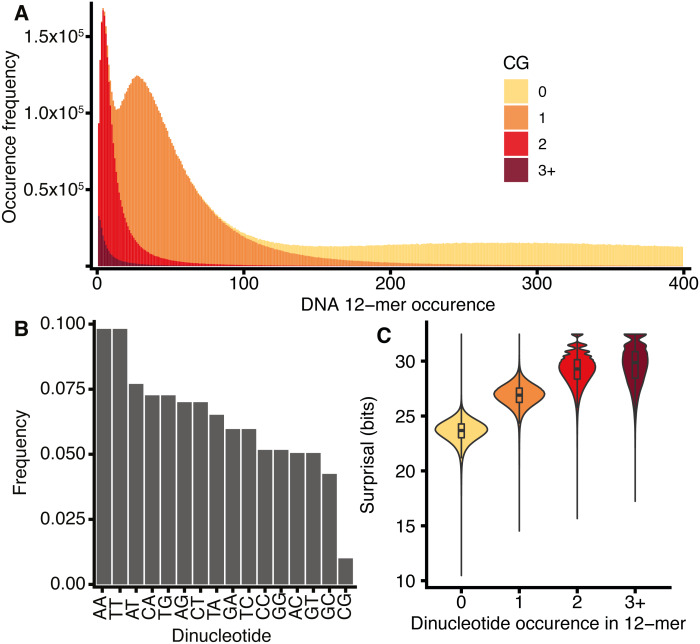
*H. sapiens* DNA *k*-mer distributions are dependent on CG content. (A) Stacked bar chart representing 12-mer occurrences for all 12-mers in *H. sapiens*, segregated according to the number of CG dinucleotides in each *k*-mer. *x*-axis: number of times a *k*-mer occurs in the genome, *y*-axis: number of distinct *k*-mers occurring at a given frequency. (B) The frequency of occurrence of the 16 dinucleotides in *H. sapiens* DNA normalised to the total number of all dinucleotides, including reverse complements. (C) Violin and box plot of surprisal distributions for 12-mers segregated by CG dinucleotide content.

The spectra of *k*-mers in the DNA sequences of several species were previously described by Chor et al. (2009). Figure 4 shows the *H. sapiens* 12-mer DNA spectrum, which reveals three peaks in *k*-mer abundance corresponding to 12-mers occurring 4, 27, and 298 times in the genome. The spectrum can be viewed as three overlapping distributions, which are largely explained by the number of CG dinucleotides that occur within each 12-mer. This behaviour is not observed for any other dinucleotides (Fig. S5).

This CG dependency was also reported by Chor et al. (2009) for *k*_*nt*_ = 8, who suggested that it is a property of tetrapod genomes as a consequence of a global repression of CG dinucleotides, as shown in Fig. 4C for *H. sapiens*. This effect is further exacerbated by the concentration of CG dinucleotides at CpG islands, represented by a small number of CG-rich, common *k*-mers. The repression of CG dinucleotides is an established feature of genome evolution and results from the high mutation rate of CG dinucleotides caused by the deamination of methylated cytosines (reviewed by Walsh & Xu (2006)). A secondary consequence of the disproportionate rarity of CG dinucleotides is that *k*-mers that contain them tend to have high surprisal (Fig. 4D).

**Figure 5:**
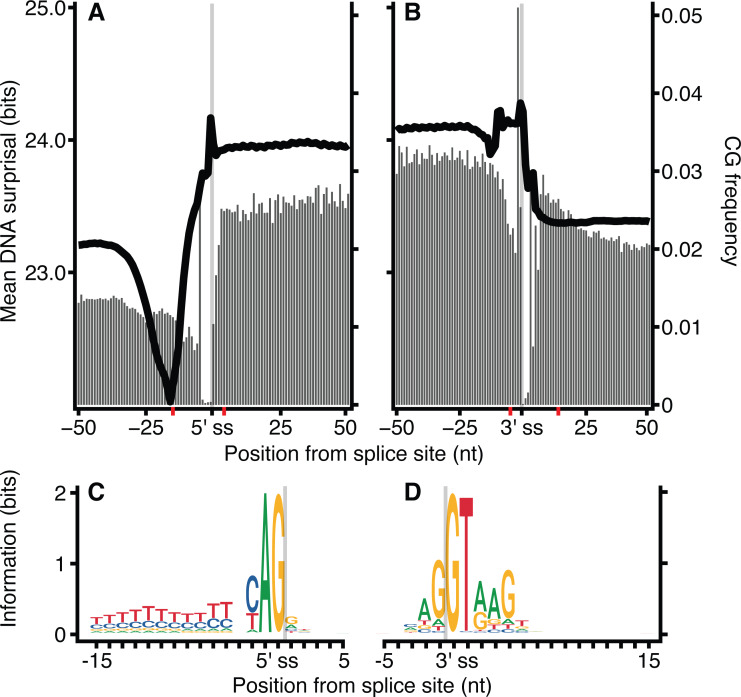
CG dinucleotide count affects *H. sapiens* exon intron boundary surprisal. (A, B) Average surprisal across exon-intron and intron-exon boundaries, as per Fig. 2 (line), and CG count frequency (bars). (C, D) sequence motifs at the 5′ and 3′ splice sites. The motif regions are identified in (A, B) by the red tick-marks on the *x*-axis.

The frequency of CG dinucleotides in proximity to exon-intron junctions was investigated for *H. sapiens* DNA sequences (Fig. 5). Similar to Fig. 2, the ‘valley’ observed in mean surprisal centred at −16 is due to the effect of the polypyrimidine tract, shown explicitly in Fig. 5C. The CG dinucleotide frequency is 2-fold enriched within exons, providing a partial explanation for the increased information content in exons compared with introns. These results are in keeping with previous reports describing high CG differential between exon and intron regions (Amit et al., 2012). However, even within exonic regions, the CG dinucleotide occurs at a much lower frequency than all other dinucleotides. No other dinucleotides show such a disproportionate difference between intron and exonic spaces (Fig. S6). However, CG dinucleotide usage is not sufficient to explain all patterns, as shown by the lack of correspondence between CG dinucleotide usage and surprisal across the polypyrimidine tract.

## Discussion

Here we describe a novel method for interrogating the genome using the self-information content of molecular sequences. The likelihood of occurrence of a *k*-mer is inversely correlated with surprisal, and hence information content. Genome-wide biological features are represented by sequences with lower surprisal because they occur more frequently and therefore tend to be encoded with common sequences. Conversely, loci with specific functions are encoded with rarer, higher surprisal sequences which contain more information. Our results also show that the CG dinucleotide is a major factor in *k*-mer surprisal. However, as shown in Fig. 5, while the CG dinucleotide is a major contributor to overall surprisal patterns, it is not the only factor at play.

**Figure 6:**
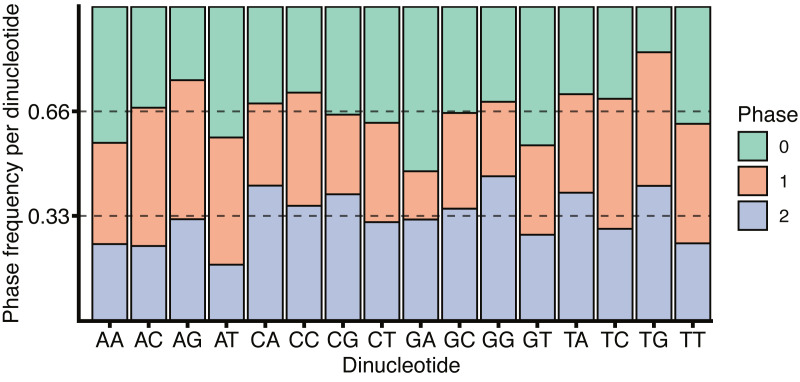
*H. sapiens* phased dinucleotide usage. Phase frequency of each dinucleotide within *H. sapiens* coding sequences split by the codon phase, phase 0 corresponds to the dinucleotide in codon positions 1 and 2, phase 1 would be codon positions 2 and 3 and phase 2 corresponds to position 3 in the upstream codon and position 1 in the downstream codon. Dotted lines are drawn at 1/3 and 2/3 representing un-skewed dinucleotide phases.

The role of CG dinucleotides in CpG islands, DNA methylation (Deaton & Bird, 2011) and the hyper-mutability of methylated cytosines (Misawa & Kikuno, 2009) are all well understood, however the variation within the coding and non-coding regions around splice boundaries is less well characterised. While the increase in CG dinucleotide usage within coding regions, as observed in Fig. 5, may be due to the necessity to encode amino acids, a remarkable feature of the codon table is that all amino acids, and all amino acid sequences, can be represented without the use of the CG dinucleotide. Thus, all codons containing a C in position 2 have complete redundancy in the 3rd base. Further, while a CG in positions 1 and 2 (i.e., CGN) all encode arginine, arginine can also be encoded by AGY (where N corresponds to any nucleotide, and Y to a pyrimidine). Finally, all amino acids are redundant in the 3rd base between pyrimidines. It is therefore surprising that exon sequences are enriched for CG dinucleotides relative to the surrounding introns, particularly given the tendency for deamination driven C to T transitions. It is tempting to speculate that CG dinucleotide retention within exons is in part driven by the need to encode additional regulatory sequences within the same coding locus. This is in keeping with Fig. 6 which shows no substantial correspondence between CG usage and coding phase.

Our method has been applied genome-wide to regions involved in the established biological process of splicing. It successfully quantifies information patterns of known splicing motifs including splice sites and the polypyrimidine tract. The method can also be used to compare information content between different types of molecular sequence, such as coding mRNAs and amino acid sequences. Importantly, surprisal patterns observed at exon-exon boundaries for nucleotide sequences are not driven by the associated amino acid information (Fig. 3) suggesting that the position of exon boundaries can be accommodated by codon redundancy. This observation makes sense since translation occurs as a subsequent biological step after splicing.

The use of *k*-mers for sequence analysis is common and simple since implementations are available that are amenable to large scale computation. Here we use *k*-mers as the basis with which to compute surprisal patterns across loci. The method makes it possible to compare loci irrespective of the sequence type or similarity, as shown with the analysis of splice boundaries (Fig. 3). Having demonstrated the utility of our model, we will next apply it to the impact of SNVs in genetic diseases.

## Conclusions

The information content of biological sequences has been modelled for many decades, but methods that rely on multiple sequence alignments are challenging when sequence divergence is large. Here, we describe a model in which a measure of the self-information content of molecular sub-sequences, *k*-mer surprisal, is used to quantify information content associated with biological features. Using splice sites as an exemplar, our model reveals clear patterns in surprisal around exon junctions that is observed consistently across a panel of evolutionarily diverse eukaryotes. Many of these patterns can be attributed to known biology, including binding motifs and patterns of dinucleotide usage. This surprisal model is complementary to existing approaches, and can be used to investigate sequence information content without the need for multiple sequence alignments.

## Code availability

The model was developed using MapReduce (http://mapreduce.sandia.gov) formulation in C++ (Plimpton & Devine, 2011). Analysis and figure plotting was performed in R using R-packages (R Core Team, 2019; Dowle & Srinivasan, 2019; Wickham, 2017; Kassambara, 2020; Neuwirth, 2014; Wagih, 2017; Bengtsson, 2020; Lawrence, Gentleman & Carey, 2009; Wickham, 2011; Charif & Lobry, 2007; Wickham, 2019). All code used in this work can be found at GitLab (https://gitlab.com/cruk-mi/genomic-kmer-surprisal-model).

##  Supplemental Information

10.7717/peerj.10063/supp-1 Supplemental Information 1 Exon-intron boundaries for varying *k* Similar to Fig. 2 with A, C, E, G, and I representing the 5′ exon-intron boundaries and B, D, F, H, and J representing the 3′ exon-intron boundaries for varying *k* = 3 (A, B), 6 (C, D), 9 (E, F), 12 (G, H) and 15 (I, J). *S. pombe* is included for reference.

10.7717/peerj.10063/supp-2 Supplemental Information 2 *H. sapiens* Exon-intron boundaries coefficient of variation Exon-intron boundary for *H. sapiens* sequences in Fig. S1. The coefficient of variation (mean/sd) for exon-intron 5′ (A) and 3′ (B) boundaries with varying *k* = 3, 6, 9, 12 and 15.

10.7717/peerj.10063/supp-3 Supplemental Information 3 Exon-exon boundaries for varying *k* Similar to Fig. 3, where DNA, coding mRNA and amino acid sequences (top to bottom) are plotted for varying *k* = 3, 6, 9, 12 and 15 (left to right). *y* axes are a constant size for each k columns, however the range is shifted dependent on the sequence type (with the exception of *k* = 15)

10.7717/peerj.10063/supp-4 Supplemental Information 4 *H. sapiens* exon-exon boundaries coefficient of variation Exon-exon boundary for *H. sapiens* sequences in Fig. S3. The coefficient of variation (mean/sd) for DNA (A), coding mRNA (B) and amino acid (C) surprisal varying by *k* = 3, 6, 9, 12 and 15.

10.7717/peerj.10063/supp-5 Supplemental Information 5 *H. sapiens* 12-mer spectrum split by dinucleotide occurrence Distribution spectra as in Fig. 4A, but with colours split by the labelled dinucleotides. Stacked bar chart representing 12-mer occurrences for all 12-mers in *H. sapiens*, segregated according to the number of each dinucleotide in each k-mer. x-axis: number of times a *k*-mer occurs in the genome, *y*-axis: number of distinct *k*-mers occurring at a given frequency.

10.7717/peerj.10063/supp-6 Supplemental Information 6 CG dinucleotide count affects *H. sapiens* exon intron boundary surprisal Similar to Figs. 5A and 5B. Exon-intron and intron-exon boundaries showing dinucleotide count frequency for each dinucleotide.

## Additional Information and Declarations

### Data Availability

The following information was supplied regarding data availability: All code is available at GitLab: https://gitlab.com/cruk-mi/genomic-kmer-surprisal-model.
