# Supplementary figures and images for: A model of k-mer surprisal to quantify local sequence information content surrounding splice regions
Source: PeerJ. 2020 Nov 4;8:e10063. doi: 10.7717/peerj.10063 (PMC7648452; doi:10.7717/peerj.10063)

*H. sapiens* *D. rerio* *S. pombe*  
*M. musculus* *D. melanogaster*

Mean DNA surprisal (bits)

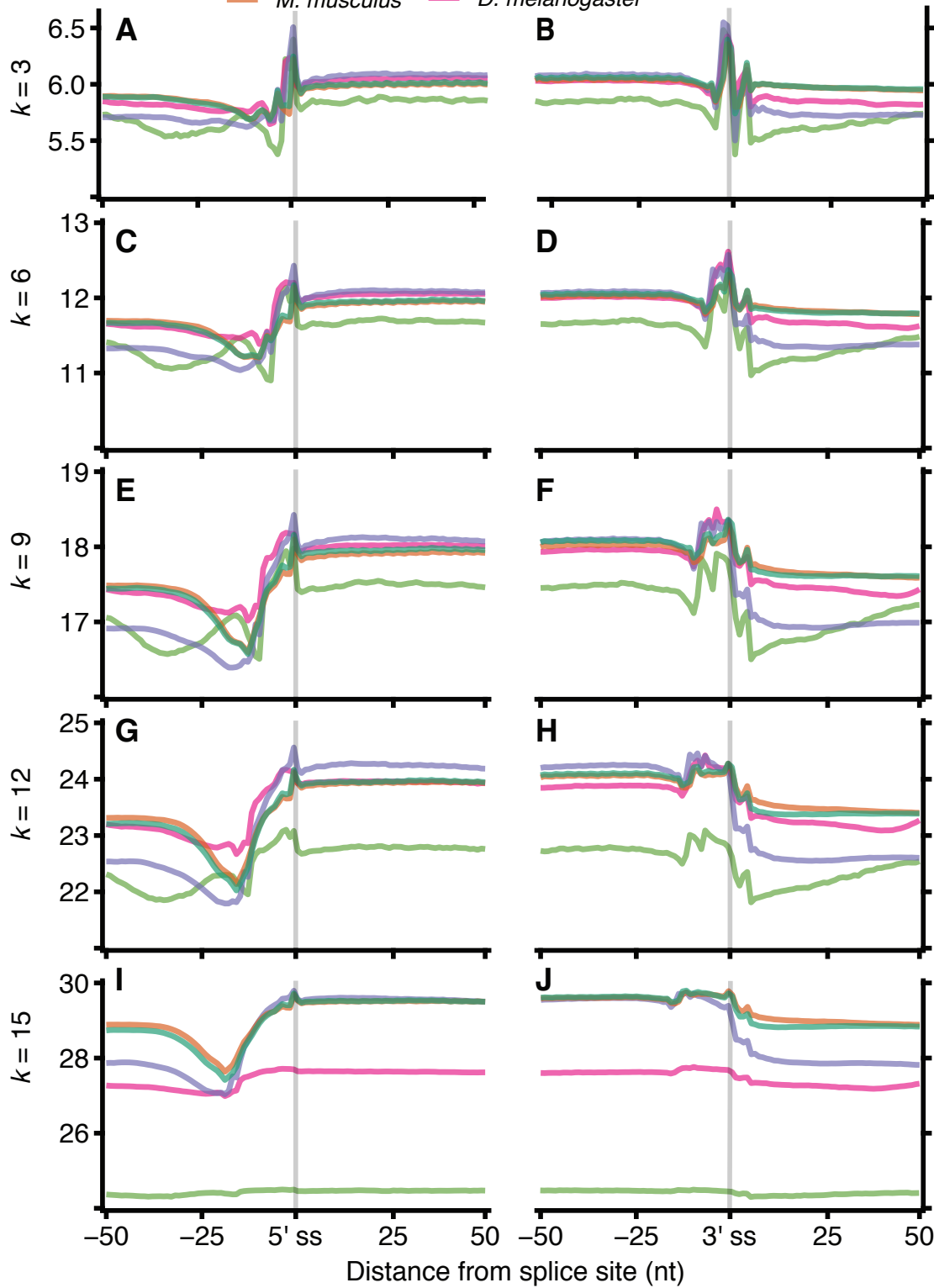

Supplement: Supplemental Information 1 — Similar to Fig. 2 with A, C, E, G, and I representing the 5′ exon-intron boundaries and B, D, F, H, and J representing the 3′ exon-intron boundaries for varying k = 3 (A, B), 6 (C, D), 9 (E, F), 12 (G, H) and 15 (I, J). S. pombe is included for reference. [file peerj-08-10063-s001.pdf]

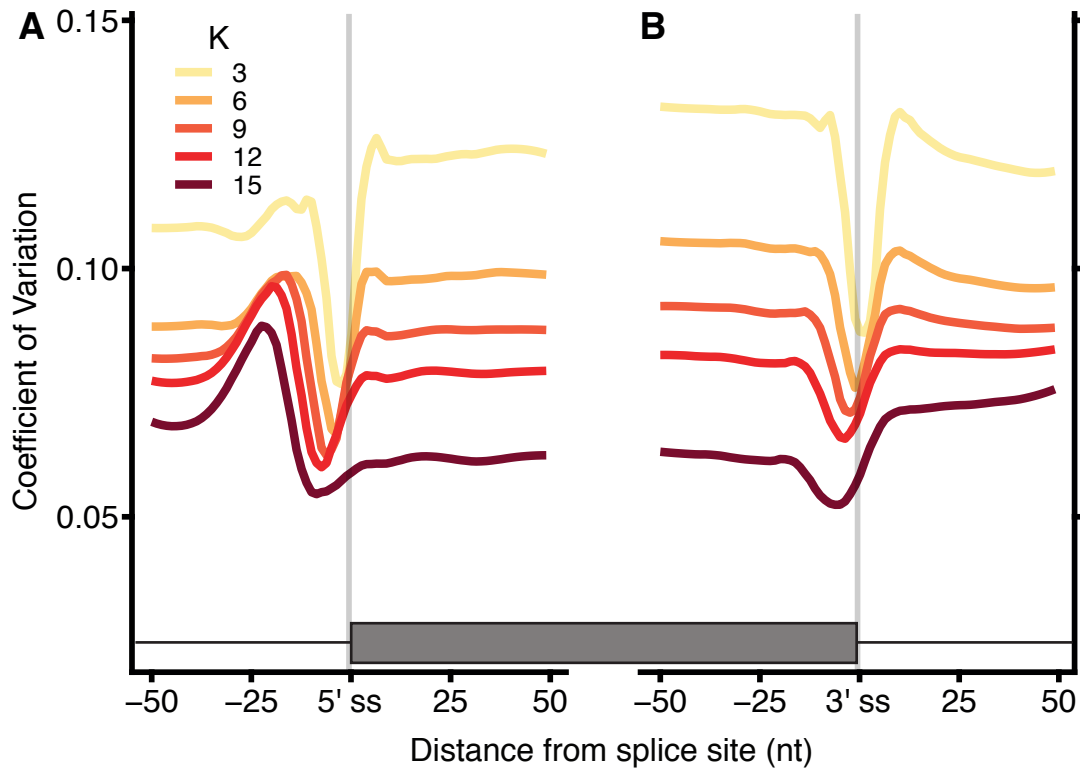

Supplement: Supplemental Information 2 — Exon-intron boundary for H. sapiens sequences in Fig. S1. The coefficient of variation (mean/sd) for exon-intron 5′ (A) and 3′ (B) boundaries with varying k = 3, 6, 9, 12 and 15. [file peerj-08-10063-s002.pdf]

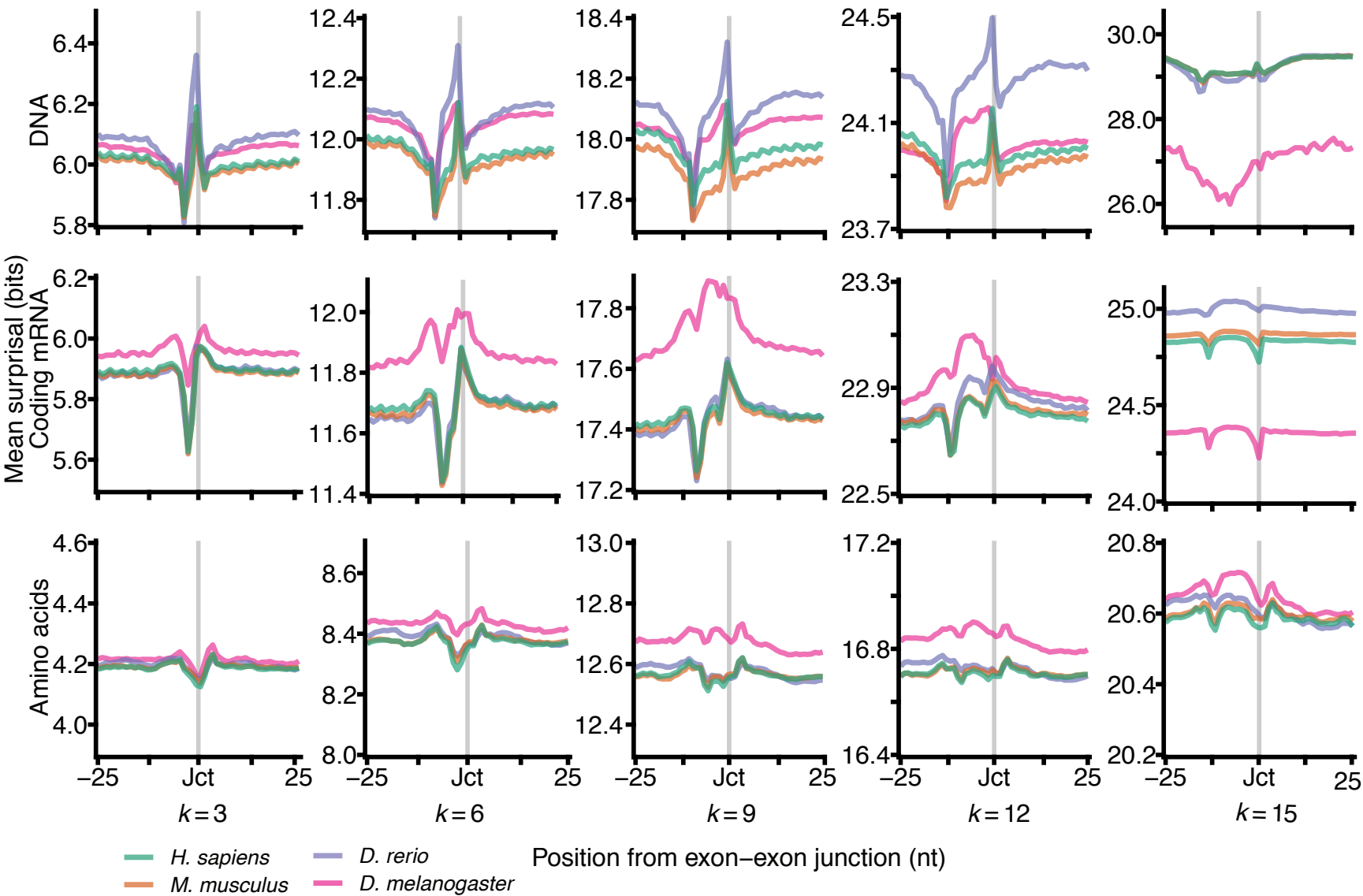

Supplement: Supplemental Information 3 — Similar to Fig. 3, where DNA, coding mRNA and amino acid sequences (top to bottom) are plotted for varying k = 3, 6, 9, 12 and 15 (left to right). y axes are a constant size for each k columns, however the range is shifted dependent on the sequence type (with the exception of k = 15) [file peerj-08-10063-s003.pdf]

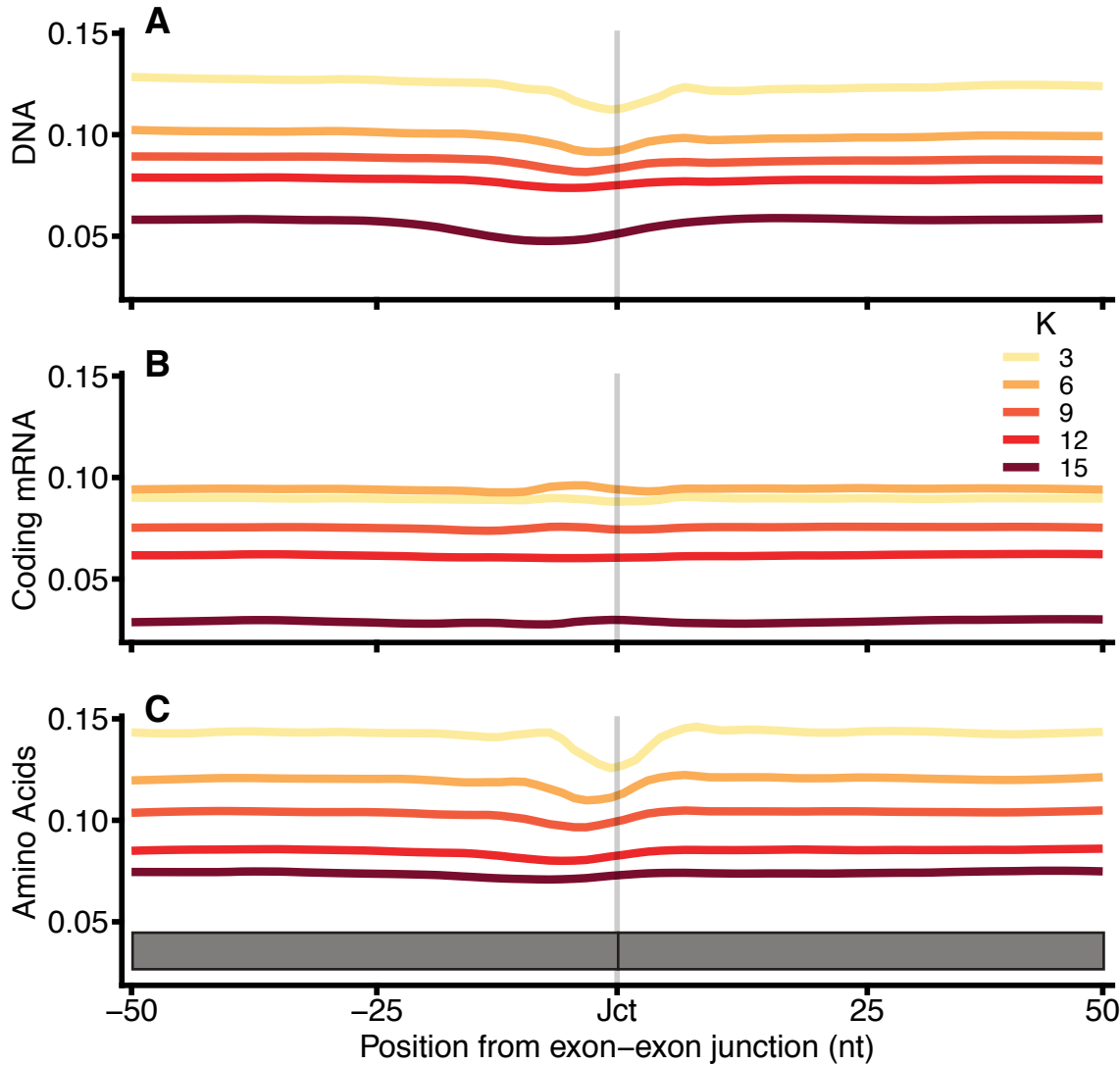

Supplement: Supplemental Information 4 — Exon-exon boundary for H. sapiens sequences in Fig. S3. The coefficient of variation (mean/sd) for DNA (A), coding mRNA (B) and amino acid (C) surprisal varying by k = 3, 6, 9, 12 and 15. [file peerj-08-10063-s004.pdf]

Dinucleotide count

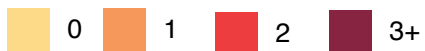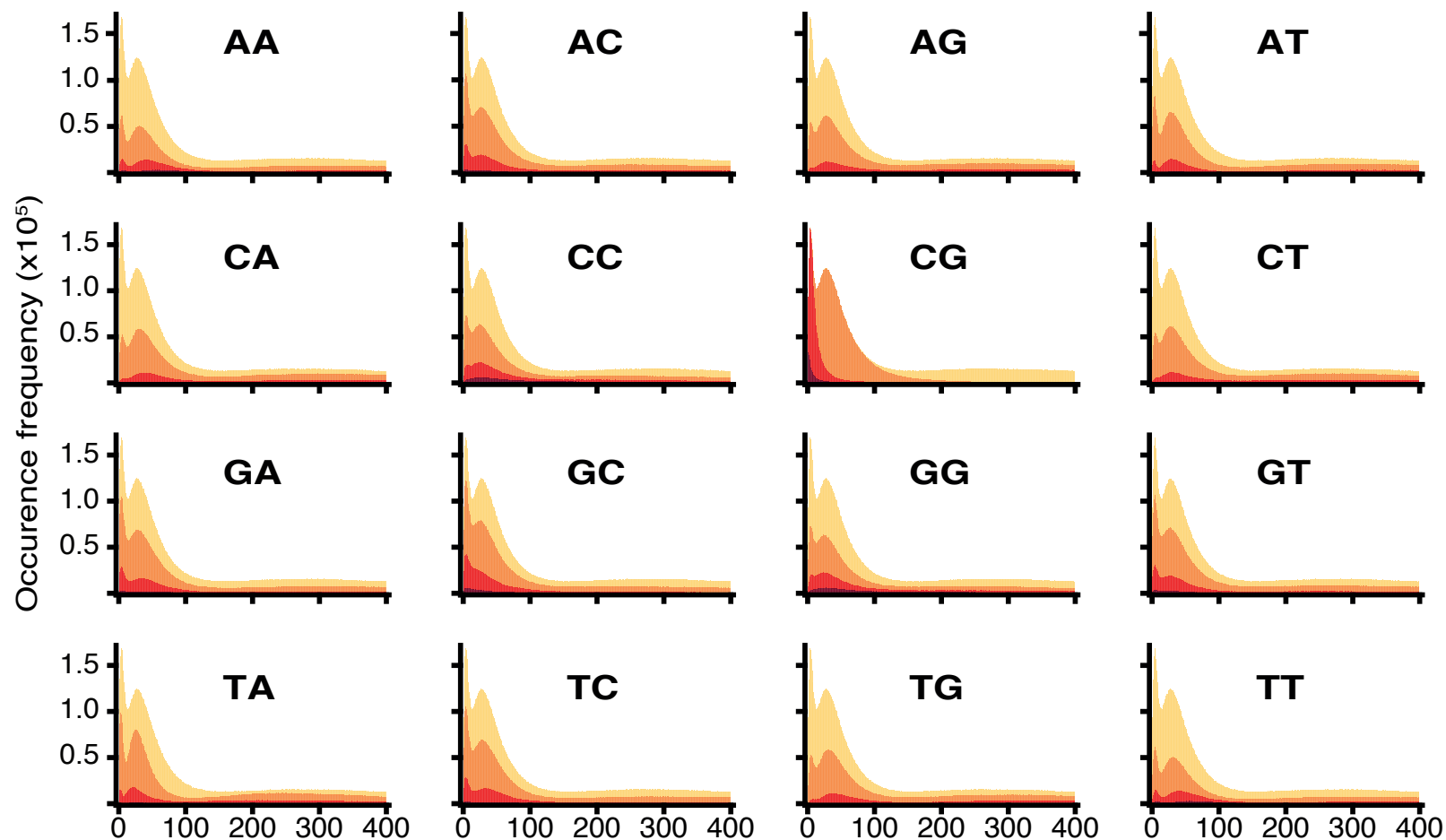

DNA 12-mer Occurrence

Supplement: Supplemental Information 5 — Distribution spectra as in Fig. 4A, but with colours split by the labelled dinucleotides. Stacked bar chart representing 12-mer occurrences for all 12-mers in H. sapiens, segregated according to the number of each dinucleotide in each k-mer. x-axis: number of times a k-mer occurs in the genome, y-axis: number of distinct k-mers occurring at a given frequency. [file peerj-08-10063-s005.pdf]

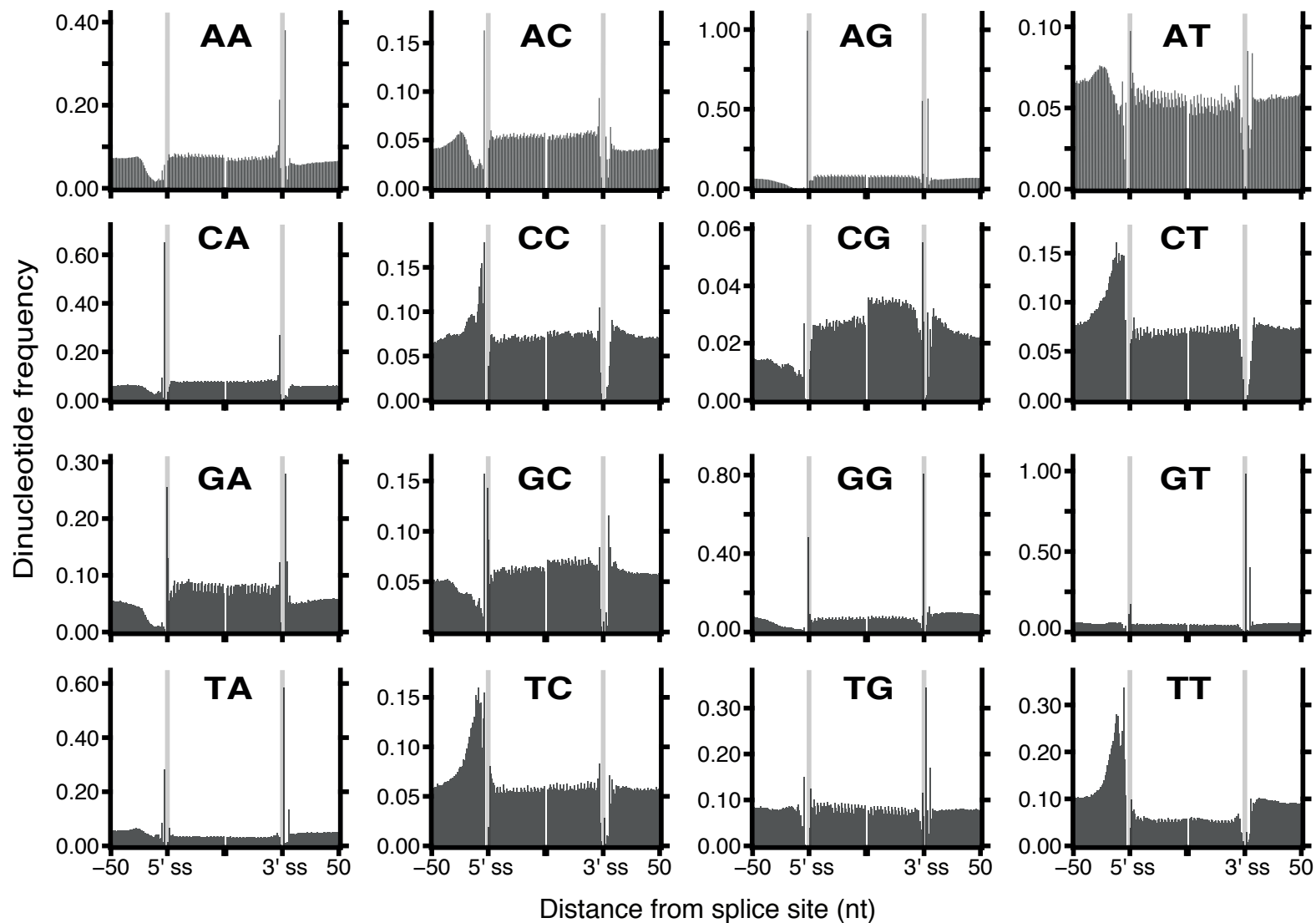

Supplement: Supplemental Information 6 — Similar to Figs. 5A and 5B. Exon-intron and intron-exon boundaries showing dinucleotide count frequency for each dinucleotide. [file peerj-08-10063-s006.pdf]
